# Four perspectives for oncological children support: the play-based approach as an integrative framework

**DOI:** 10.3389/fpsyg.2026.1730525

**Published:** 2026-03-16

**Authors:** Giulia Perasso, Marco Romeo, Luminita Andreescu, Paola Coccia, Giulia Palego, Paulina Pérez-Duarte Mendiola

**Affiliations:** 1 University of Genoa, Genoa, Italy; 2 Istituto Oncologico per il Sostegno Integrato e la Ricerca Multidisciplinare “Semper Onlus”, Fano, Italy; 3 Associazione Nazionale Volontari Lotta contro i Tumori (ANVOLT), Trieste, Italy; 4 Presidio Ospedaliero G. Salesi, Ancona, Italy; 5 Azienda Ospedaliero Universitaria delle Marche, Ancona, Italy; 6 Great Ormond Street Hospital for Children NHS Foundation Trust, London, United Kingdom

**Keywords:** child-centered care, pediatric oncology, play specialism, psychosocial support, therapeutic play

## 1 Introduction

Pediatric hospitalization often evokes specific fears in children (e.g., mutilation, pain, death, separation from family) (Coyne, 2006) and power imbalance (Bricher, 2000). In hospital settings, critical decisions are generally made by adults while children are frequently excluded from medical discussions, perceiving themselves reduced to clinical labels (Pérez-Duarte Mendiola, 2020, 2024). This can lead to anxiety, specific phobias, depressive symptoms, or behavioral regression (Chambers, 1993). Nevertheless, the psychological impact of long-term hospitalization is often underestimated (Koukourikos et al., 2015). These consequences are especially salient for children with oncological and hemato-oncological diagnoses (Obaid, 2015), especially in terms of pervasive preoccupation with illness and death, social withdrawal, and fear of losing independence.

Nevertheless, children with cancer are not passive victims of their circumstances. Within a biopsychosocial framework (Engel, 1978), their adaptive coping skills are shaped by multiple factors, including age and stage of illness at diagnosis, parents' coping strategies, family background (e.g., socioeconomic status, culture, religion, optimism, resilience, social support), personal experience (e.g., previous death of a grandparents or a pet), and opportunities for peer interaction (Kupst et al., 1984; Varni et al., 1994; Bluebond-Langner, 1978). Pediatric oncology patients often exhibit resilience and adaptive adjustment (Phipps, 2007; Phipps et al., 2014). Recognizing the inherent resources of children with cancer is essential for healthcare professionals, as it enables the use of psychosocial interventions to support and enhance wellbeing (Perasso and Ozturk, 2022). Literature emphasizes that interventions such as psychotherapy, family therapy, art activities, and play-based programs are crucial for the child's psychosocial adjustment (Coughtrey et al., 2018), but also for the wellbeing of parents and healthcare professionals (Ong et al., 2025).

At the same time, differences among healthcare professionals in their training, orientation, and strategies highlight the need to reflect about the different healthcare profiles that may arise in pediatric oncology (Louwen et al., 2023). In this opinion paper, we present four different healthcare perspective to oncological children support. Also propose a unifying framework grounded in play specialism (Perasso and Ozturk, 2022).

### 1.1 The emotional-relational perspective

Emotional-relational professionals in pediatric oncology focus on integrating clinical expertise with deep emotional attunement and relational sensitivity. Central to their approach is the cultivation of compassion, conceptualized as sympathy toward another in distress and the desire to help (American Psychological Association, 2018). Strauss et al. (2016) further break down compassion into five core elements: (1) recognizing another's suffering; (2) understanding the universal nature of pain; (3) feeling for the suffering of others; (4) tolerating uncomfortable emotional experiences; (5) and motivation to alleviate distress. These elements form the foundation of compassionate care frameworks, such as the Compassionate Nursing Care Model (CNCM), emphasizing the importance of empathy and proactive engagement with patients' emotional and physical needs (Ghafourifard et al., 2022).

In pediatric oncology, compassionate care is crucial, especially in end-of-life situations. It enables professionals to establish meaningful emotional connections with both patients and family caregivers, facilitating preparation for—and acceptance of—death while addressing complex emotional and existential needs (Skorpen Tarberg et al., 2020).

The Pediatric Compassion Model (Sinclair et al., 2020, 2021) identifies four interrelated dimensions: (1) beneficence, as addressing suffering can evoke virtues such as love, kindness, and prudence; (2) human relating, emphasizing emotional resonance and genuine communication with patients and families; (3) seeking to understand, leading to comprehension of each individual's unique suffering and co-creation of care plans; and (4) attending to needs, involving timely, kind, and responsive actions toward patient necessities. In pediatric oncology, this approach incorporates empathy, altruism, respect for cultural and religious backgrounds, and sensitive facilitation of end-of-life care, including enabling parents' and—in some cases—siblings' presence at the child's bedside (Ghaljeh et al., 2024).

In essence, emotional-relational professionals in pediatric oncology function as caregivers who balance clinical expertise with humanistic and ethical virtues. Their work demonstrates that optimal care extends beyond technical interventions to encompass the emotional dimensions of the child's experience. They actively recognize and respond to suffering (Strauss et al., 2016), foster empathic and compassionate connections with both patients and family caregivers (American Psychological Association, 2018; Skorpen Tarberg et al., 2020), and maintain sensitivity to cultural, spiritual, and individual needs (Ghaljeh et al., 2024). By engaging in human-relational practices and co-creating care plans based on deep understanding of each child's unique circumstances, they build trust, alleviate distress, and support the child's and family's coping capacity (Sinclair et al., 2020, 2021).

### 1.2 The communicative-systemic perspective

These professionals integrate clinical expertise with awareness of relational dynamics among patients, families, and healthcare teams. These specialists adopt a family-centered care (FCC) approach, recognizing parents and eventually other caregivers (grandparents or older siblings) as primary experts on the child and actively involving family members in healthcare decisions (Kuo et al., 2012). FCC fosters open information sharing, respect for differences, collaboration, and negotiation with the family. FCC also stresses involving children in healthcare decisions (Coyne and Gallagher, 2011).

In pediatric oncology, communication is central to building trust, supporting family self-management in difficult circumstances, managing uncertainty, and responding to emotional needs (Sisk et al., 2020). Communication also serves to sustain hope and validate deep emotions for both children and their families (Sisk et al., 2020). Young patients express a strong desire to participate in discussions about their illness, treatment, and prognosis, although their informational needs may change over time (Brand et al., 2017). Therefore, systemic-communicator professionals routinely assess the child's desired level of involvement and adjust communication strategies according to developmental stage, neurodivergence, disabilities, and other circumstances (Oulton et al., 2016; Wray et al., 2025).

A systematic review of children's experiences with clinician communication during pediatric cancer care highlighted key themes (Lin et al., 2020): children often feel invisible, powerless, or intimidated by adult authority. They also fear receiving devastating news and they may experience burdens from external expectations. Effective communication fosters empowerment and promotes assertive agency, enabling children to develop self-management skills. Thus, medical communication should actively involve the child and not be solely parent-centered.

These professionals create feedback loops that strengthen trust, information exchange, and patient agency (Kuo et al., 2012; Sisk et al., 2020; Brand et al., 2017; Lin et al., 2020; Feraco et al., 2016). In essence, systemic-communicator healthcare professionals act as relational mediators, bridging the gap between medical expertise and family knowledge while supporting the child's voice, autonomy, and coping capacities.

### 1.3 The normalizing-creative perspective

Healthcare professionals can use play and creativity to normalize hospital life and foster creativity (Hubbuck, 2009; Perasso et al., 2021; Neves Calventi et al., 2024). These professionals, whom we may term “Normalizing-Creatives,” employ play-based and expressive strategies aimed at creating continuity between home life and the hospital environment. With these strategies they help children maintaining a sense of childhood despite the restrictions imposed by illness and treatment (Burns-Nader and Hernandez-Reif, 2016). They help children to become familiar with hospital instruments and procedures, reducing anxiety toward this experience (Sutton-Smith, 1999; Cameron and Patte, 2018). Through these strategies, children gain a greater sense of control and safety, while specialists facilitate emotional expression and cooperation during treatments (Pérez-Duarte Mendiola, 2024).

These professionals' education combines psychological, pedagogical, and medical knowledge, enabling them to design individualized play interventions tailored to the child's age and clinical condition (Lookabaugh and Ballard, 2018; Metzger et al., 2013). Play-based interventions can reduce anxiety, stress, and pain, enhance coping skills, and support emotional and social development during hospitalization (Gill, 2010; Moore et al., 2015; Perasso et al., 2021, 2025), also in difficult times like the Covid-19 pandemic (Tsamakis et al., 2020).

In this scenario, normalizing-creatives healthcare professionals employ creativity to increase children's resilice. Their work goes beyond entertainment, actively contributing to children's psychological well-being and adaptation, emphasizing the value of integrated approaches that consider the complexity of the child as a whole, not merely as a clinical patient (Perasso et al., 2021).

### 1.4 The structured-clinical perspective

Structured-clinical professionals adopt an evidence-based psycho-oncological perspective. A foundational framework guiding their practice is the adaptive style paradigm, which conceptualizes children with cancer as resilient individuals who often adopt a repressive style to manage stress while maintaining emotional stability (Weinberger et al., 1979; Phipps, 2007). Structured-clinical healthcare professionals therefore view children as active agents with significant potential for wellbeing, rather than as passive recipients of medical care.

In the practice, these professionals employ targeted techniques such as mindfulness, relaxation exercises, and meditation to reduce anxiety, distress, and depressive symptoms (Zarou et al., 2022). They understand that psychological support can facilitate the development of adaptive coping mechanisms across different ages and stages of illness, ensuring that each child is equipped to manage both immediate and long-term stressors (Kupst et al., 1984). Weinstein and Henrich (2013) identified key strategies in clinical approach including: explaining procedures to educate and prepare children, offering emotional support by listening and comforting, and utilizing distraction techniques to mitigate stress.

Structured-clinical healthcare professionals exemplify a model of pediatric oncology care in which psycho-oncological knowledge, technical expertise, and evidence-based strategies converge. By applying the adaptive style paradigm (Weinberger et al., 1979; Phipps, 2007; Phipps et al., 2014) and leveraging structured psychological techniques (Kupst et al., 1984; Zarou et al., 2022; Weinstein and Henrich, 2013), they facilitate children's coping and enhance their resilience.

### 1.5 Play Specialism: an integrative perspective

Optimal pediatric oncology care requires a multidimensional approach that acknowledges the contributions of the emotional- relational, communicative-systemic, normalizing-creative, and structured-clinical perspectives. Play Specialism can embody a unifying role, integrating all these approaches into a coherent, child-centered framework of care.

The Play Specialist (PS) or Health Play Specialist (HPS) combines relational sensitivity, communication mediation, structured clinical interventions, and therapeutic play, offering children both a sense of continuity and agency in the hospital setting (Beickert and Mora, 2017). Through compassionate presence, PS recognize suffering, validate emotions, and foster trust and coping, while mediating between families and healthcare teams to enhance participation and shared decision-making (Sinclair et al., 2020; Kuo et al., 2012). Play interventions normalize hospitalization, reduce anxiety, and allow children to explore illness-related experiences safely (Sutton-Smith, 1999; Cameron and Patte, 2018; Romito et al., 2021). These interventions are not mere recreation: as recognized by Article 31 of the UN Convention on the Rights of the Child and the European Association for Children in Hospital, play is a fundamental right and a vital indicator of developmental and emotional wellbeing (Lundy, 2012; European Association for Children in Hospital, 1988; Koukourikos et al., 2015).

Historically, PS have been recognized as psycho-pedagogical professionals rather than recreational staff (Plank, 1962; Bergmann, 1965; Frauman and Gilman, 1989; Francischinelli et al., 2012; Brooks, 1970). Modern PS practice operationalizes these principles, using age- and diagnosis-specific interventions to support coping, adherence to treatment, and emotional regulation (Burns-Nader and Hernandez-Reif, 2016; Barry, 2008; Tanaka et al., 2010). PS interventions improve pain management, reduce sedation needs, increase compliance with medical procedures, and support palliative care, benefiting children, families, and healthcare teams (Gill, 2010; Li et al., 2016; Basak et al., 2019; Metzger et al., 2013; Grissom et al., 2016). Multidisciplinary training is central to the PS role (Harvey, 1984; Lookabaugh and Ballard, 2018; Beickert and Mora, 2017). Certification programs—including Healthcare Play Specialist in the UK and Certified Child Life Specialist in the US—standardize practice, fostering consistent quality of care across settings (Hart-Spencer and Griffiths, 2015; Perasso et al., 2021).

From a clinical implementation perspective, Play Specialism may be operationalized through the integration of Play Specialists within multidisciplinary pediatric oncology teams. In practice, this may involve early referral at diagnosis, participation in treatment preparation, and collaboration with physicians, nurses, psychologists, and families to identify moments of increased emotional or procedural distress (Abdelkhalik et al., 2024). Structured play-based interventions can be incorporated into routine care pathways, such as procedural preparation, symptom management, and transitional phases across the illness trajectory (Ortiz La Banca et al., 2020). Embedding Play Specialists within standard clinical workflows may facilitate continuity of psychosocial support, promote child participation in care, and strengthen communication between children, families, and healthcare professionals.

The integrative model offered by PS provides tangible benefits: improved emotional adaptation, strengthened family collaboration, reduced anxiety, enhanced coping skills, and continuity of psychological support between hospital and home (Perasso et al., 2021; Moore et al., 2015; Romito et al., 2021). By uniting relational, communicative, creative, and clinical expertise, PS practice addresses the full spectrum of children's psychosocial needs and positions play as a therapeutic, rights-based intervention.

## 2 Conclusion

Each of the four perspectives described provides essential support to children with cancer, addressing diverse psychological, developmental, and relational needs. However, no single approach is sufficient. Integration is crucial, and the Play Specialist embodies this multidimensional role, merging empathy, communication, creativity, and clinical expertise. Creating a compassionate, supportive, and child-centered hospital environment will enable us—or bring us closer—to meeting children's needs holistically.
